# Uridine Diphosphate Promotes Rheumatoid Arthritis Through P2Y6 Activation

**DOI:** 10.3389/fphar.2021.658511

**Published:** 2021-04-19

**Authors:** Hongxing Wang, Hui Wu, Kehua Fang, Xiaotian Chang

**Affiliations:** ^1^ Medical Research Center of the Affiliated Hospital of Qingdao University, Qingdao, China; ^2^ Department of Clinical Laboratory, Qilu Hospital, Shandong University, Jinan, China; ^3^ Qingdao Engineering Technology Center for Major Disease Markers, Qingdao, China

**Keywords:** uridine diphosphate, rheumatoid arthritis, P2Y6 receptor, MRS2578, LC-MS

## Abstract

**BACKGROUND:** Uridine diphosphate (UDP) is an extracellular nucleotide signaling molecule implicated in diverse biological processes via specific activation of pyrimidinergic receptor P2Y, G Protein-Coupled, 6 (P2Y6). There is very little knowledge about the function and mechanism of UDP in rheumatoid arthritis (RA).

**METHODS:** This study used a quasi-targeted liquid chromatography-mass spectrometry (LC-MS) approach to investigate the unique expression of metabolites in RA synovial fluids (SF) (*n* = 10) with samples from osteoarthritis (OA) as controls (*n* = 10). RA fibroblast-like synoviocytes (FLSs) were collected from synovial tissues (*n* = 5) and cultured with UDP or MRS2578, a P2Y6 antagonist, and FLSs from OA were used as controls (*n* = 5). Rats with collagen-induced arthritis (CIA) were injected with UDP, MRS2578 or both (*n* = 9 for each group). P2Y6 expression was examined using real-time PCR, Western blotting and immunohistochemistry. Cell proliferation, apoptosis and migration of RA FLSs were measured using CCK-8 assay, real-time cell analysis, flow cytometry, wound healing assay and Transwell assay, respectively. The UDP levels in the culture medium, synovial fluid (*n* = 36) and peripheral blood (*n* = 36) of RA and CIA rats were measured using a Transcreener UDP Assay. Levels of proinflammatory cytokines were measured using a flow assay. Interleukin-6 (IL-6) levels were measured using ELISA and flow.

**RESULTS:** LC-MS analysis detected significantly increased UDP levels in RA SF compared with OA SF, and the level was positively correlated with anticyclic citrullinated peptide (anti-CCP) and rheumatoid factor (RF)levels in RA. The increased UDP concentration was verified in the blood and synovial fluids of RA patients compared with samples from OA patients and healthy volunteers, respectively. UDP stimulated cell proliferation, migration and IL-6 secretion in RA FLSs and inhibited their apoptosis in culture, and MRS2578 inhibited these effects of UDP. UDP injection accelerated CIA and stimulated IL-6 production rather than other proinflammatory cytokines in the rat model, but simultaneous injection of MRS2578 suppressed these effects and alleviated CIA. P2Y6 expression was increased in RA and CIA synovial tissues.

**CONCLUSION:** These results suggest that UDP is highly expressed in RA and stimulates RA pathogenesis by promoting P2Y6 activities to increase IL-6 production.

## Background

Rheumatoid arthritis (RA) is a chronic and systemic autoimmune inflammatory disease (Smolen et al., 2016). Synovial fluid (SF) accumulates extensively in synovial junctions in individuals with RA. SF directly contacts the joint; therefore, it reflects the pathological state of the synovium and disease activity. The present study used metabolomics as a high-throughput approach to analyze changes in small molecular metabolites in SF samples from RA patients. We collected SF samples from RA patients and osteoarthritis (OA) patients. Quasi-targeted liquid chromatography-mass spectrometry (LC-MS), an advanced metabolomics technique that was developed in recent years, was applied to screen the unique expression of metabolites in RA SF. After metabolomic analysis, uridine diphosphate (UDP) was found to be present at a high level in RA SF, and its level was strongly correlated with anticyclic citrullinated peptide (anti-CCP) and rheumatoid factor (RF) levels in RA.

UDP molecules comprise a pyrophosphate, a ribose and a uracil group with the pyrophosphate esterified to the C5 carbon of the sugar moiety. UDP is an important extracellular nucleotide signaling molecule (Boeynaems et al., 2005) implicated in diverse biological processes via specific activation of the metabotropic pyrimidine and purine nucleotide receptor (P2Y receptor) subtype P2Y6 (Koizumi et al., 2007; Parandeh et al., 2008; El-Tayeb et al., 2011). Previous studies have shown that extracellular UDP is released from damaged or stressed cells to promote innate immune responses (Zhang et al., 2011; Li et al., 2014). However, the expression and function of extracellular UDP in RA remain unknown. In this study, we not only detected high levels of UDP in RA SF but also investigated the role of UDP/P2Y6 signaling in RA using cultured RA fibroblast-like synoviocytes (FLSs) and a rat model of collagen-induced arthritis (CIA). Abnormal FLS activities are the main characteristic of RA pathogenesis. FLSs have a major effect on RA mediated by their aggressive proliferation and production of proinflammatory cytokines such as interleukin-6 (IL-6) (Nanki et al., 2001; Nagatani et al., 2007; Wei et al., 2012; Stephenson et al., 2018; Yu et al., 2018). Diisothiocyanate (MRS2578), with an IC50 value of 37 nM, is a powerful antagonist of the nucleotide receptor P2Y6 (Mamedova et al., 2004). We treated RA FLSs and CIA rats with UDP and MRS2578 to observe the potential changes in synovial cell proliferation, apoptosis and migration, CIA-related joint inflammation and tissue destruction, and cytokine production. This study found high UDP level and investigated the role and regulatory mechanism of UDP in RA.

## Methods

### Collection of Human RA Synovial Fluids and Synovial Tissues

RA was diagnosed according to the American College of Rheumatology/European League Against Rheumatism (ACR/EULAR) classification criteria (Arnett et al., 1988; Aletaha et al., 2010). RA synovial membrane tissues (*n* = 10) were collected from the patients during knee joint arthroscopic synovectomy. The clinical information of the patients who provided synovial tissues and synovial fluids is shown in Supplementary Table S1. Human blood (*n* = 36) and synovial fluids (*n* = 36) were collected from patients with RA. The use of samples for research was approved by The Ethics Committee of The Affiliated Hospital of Qingdao University (Approval number: 20190302). The Clinical information of the patients donating blood and synovial fluids which is related to different patients/subjects is shown in Supplementary Table S2. All patients and healthy volunteers who joined this study provided informed consent.

### Metabolomic Profiling of Synovial Fluids

Synovial fluids from patients with RA (10 patients; Patient No. R41 to No. R50) and OA (10 patients; Patient No. O41 to No. O50) were injected into an ACQUITY UPLC I-Class system (Waters Corporation, Milford, United States) through a BEH amide column (100 mm × 2.1 mm i.d., 1.7 m; Waters, Milford, United States) and analyzed in a VION IMS QT mass spectrometer (Waters Corporation, Milford, United States). The LC flow rate was 0.4 ml/min with solvents A [a mixture of acetonitrile and 10 mmol ammonium acetate (pH = 9) (90/10%, volume/volume)] and B [10 mmol ammonium acetate (pH = 9)]. The sample volume was 3 μL, the column temperature was held at 45°C, and the elution gradient of solvent B was as follows: 0 min, 5%; 1.5 min, 25%; 10 min, 90%; 13 min, 90%; 13.5 min, 5% and 14.5 min, 5%. Data acquisition was performed in full scan mode (over a m/z range of 50–1,000), and the scan time was 0.1 s. The capillary voltage was 1.0 kV, and the sampling cone voltage was 40 V. The source temperature was 120°C. The desolvation temperature was 550°C, and the flow rate of the desolvation gas was 900 L/h. To assess the stability of the system, quality control (QC) samples were injected at regular intervals throughout the analysis operation.

Volcano plots were generated by analyzing the fold change (FC) and *p* values from the *t*-test results and the variable importance in projection (VIP) scores from orthogonal partial least squares discriminant analysis (OPLS-DA). Metabolites meeting the criteria of VIP>1, FC > 2 or <0.5 and *p* < 0.05 were defined as differentially expressed metabolites (DEMs).

### Weighted Gene Coexpression Network Analysis

WGCNA was performed with R software (v3.6.1). The correlation coefficients of all metabolites were calculated according to their expression values, and a soft thresholding power (*β* = 8) was selected. The coexpression similarity was mapped into a weighted undirected network and topological overlap matrix (TOM). The dynamic tree cut algorithm was used to cluster metabolites into modules. The following parameters were used: MaxBlockSize, 6,000; TOMType, unsigned; and minModuleSize, 30.

### Establishment of Collagen-Induced Arthritis in Rats

Six-week-old male Sprague-Dawley (SD) rats were purchased from JNPY Laboratory Animal Co., Ltd. (Jinan, China). The animal study protocols complied with the Guide for the Care and Use of Laboratory Animals (Bayne, 1998) and were approved by the Experimental Animal Care and Ethics Committee of The Affiliated Hospital of Qingdao University (approval number: 20190302). These rats were randomly divided into a PBS treatment group (containing 1% DMSO, *n* = 9), a UDP treatment group (10 mg/kg, *n* = 9), an MRS2578 treatment group (3 mg/kg, *n* = 9), and a UDP (Sigma-Aldrich, Germany) and MRS2578 (MedChemExpress, United States) treatment group (*n* = 9). UDP and MRS2578 were dissolved in PBS (containing 1% DMSO). Bovine type II collagen (Chondrex, United States) was mixed with complete Freund’s adjuvant (Sigma-Aldrich, Germany) at a proportion of 1:1 and fully emulsified. The initial immunization was performed by intracutaneous injection at the tail root. Three weeks later, a booster immunization was administered using a mixture of bovine type II collagen and incomplete Freund’s adjuvant (Sigma-Aldrich, Germany) by the same route as the initial immunization at a proportion of 1:1. The injections were for a total of six consecutive administrations (twice weekly). Rats were sacrificed 20 days after the first UDP injection (3 days after the last UDP injection). The inflammation curve showing the degree of joint swelling with time was constructed. We decided to use doses of UDP and MRS2578 based on previously published literature (Koizumi et al., 2007; Vieira et al., 2011). The animals were housed in specific pathogen-free (SPF) conditions. The rats were fed a commercial pelleted diet (JNPY Laboratory Animals, Jinan, China). The animals were kept in a room with a controlled 12 h light-dark cycle under controlled temperature, humidity of 50–70% and controlled bacterial conditions.

Rats were anesthetized by intraperitoneal injection of 3% sodium pentobarbital, and blood samples were collected from the inferior vena cava. In addition, the articular cavity of the rats was collected and washed three times with PBS (1 ml) to obtain synovial fluids. These rats were euthanized with lethal doses of ketamine and xylazine, and the joint tissues within 0.5 cm of the knee joints were collected.

### Evaluation of Joint Inflammation

The inflammation curve of the joint swelling degree was constructed according to the size of the hind paws, which was measured every other day using Vernier calipers according to the method we mentioned before (Wang et al., 2019). Bone erosion and cartilage destruction in the ankle joint and knee joint were assessed by X-ray imaging (75 kV, 195.3 mA) before sacrifice. The joint tissue within 0.5 cm of the knee joint of rats was collected, fixed with 4% paraformaldehyde and embedded in paraffin. Hematoxylin-eosin staining was used to examine the pathological changes in joint tissues. According to clinical and histological evidence, the disease score was calculated as follows: 0 = normal joint; 1 = local swelling and/or erythema without histological damage; 2 = swelling and/or rigidity of the whole paw without histological damage; 3 = limb deformity with reversible histological damage; and 4 = limb deformity accompanied by permanent histological damage such as bone or cartilage erosion. The above protocol was designed based on other studies. We mentioned this method in our previous work (Wang et al., 2019).

### Isolation and Culture of Human Synovial Fibroblast Cells

Synovial tissues from patients with RA (Patient No. R41 to No. R50, *n* = 10) or OA (Patient No. 41 to No. O50, *n* = 10) were minced into small pieces and digested for 4 h at 37°C and 5% CO_2_ in 3 ml of DMEM containing 4% type II collagenase (Solarbio, China) until the tissue pieces were dispersed into a cell suspension. The cell suspension was filtered through a 70 μm cell strainer and resuspended in DMEM containing 10% FBS. Synovial fibroblast cells were incubated at 37°C in a humidified incubator containing 5% CO_2_. Cells that passed for 3–8 generations were used in subsequent experiments.

### Measurement of UDP Content

Peripheral blood samples from healthy donors (No. H1 to No. H36, *n* = 36) and RA patients (Patient No. R1 to No. R36, *n* = 36) were collected into pyrogen-free and endotoxin-free test tubes with anticoagulants. Synovial fluids from OA (Patient No. O1 to No. O36, *n* = 36) or RA patients (Patient No. R1 to No. R36, *n* = 36) were added to an equal volume of PBS. Rat synovial fluids and peripheral blood were collected as described above. These samples were centrifuged at 1,000 ×g for 20 min at 4°C, and the supernatant was carefully collected. The UDP content in the samples was measured using a Transcreener UDP Assay (BellBrook Labs, United States) via a fluorescence polarization readout according to the manufacturer’s protocol. A 15 μL mixture of reagents, including 8 nm UDP^2^ antibody-Tb, 1× Stop & Detect Buffer C and UDP HiLyte647 Tracer, was mixed with 5 μL of each sample in a 96-well plate. The plate was incubated for 1.5 h at room temperature and analyzed in a FlexStation^®^ 3 Multimode Plate Reader (Molecular Devices, United States). The concentration of UDP was calculated by the standard curve prepared with standard UDP solution before analysis.

### Measurement of Cytokine Concentrations in Blood and Culture Medium

RA synovial fibroblast cells (Patient No. R41 to No. R45, *n* = 5) were isolated and seeded in 96-well plates at a density of 3 × 10^4^ cells per well and incubated overnight. UDP was dissolved in PBS (containing 0.1% DMSO). Cells were incubated with UDP at a final concentration of 100 μM for 24 h. The supernatants were collected after centrifugation at 1,000 × g for 20 min. The IL-2, IL-4, IL-6, IL-10, TNF-α and IFN-γ concentrations in the supernatants were quantified using a Human Th1/Th2 Subgroup Detection Kit (CellGene, China). In brief, antibodies specific for IL-2, IL-4, IL-6, IL-10, TNF-α and IFN−γ were conjugated to fluorescence-encoded beads, and beads with biotinylated detection antibodies were mixed with the samples. Streptavidin-PE was added, and the mixture was incubated with shaking for 2 h at room temperature. The beads were washed and then analyzed in a NovoCyte D2040R flow cytometer (ACEA Biosciences, United States). The data were analyzed using FlowJo software (Tree Star, United States).

### Measurement of IL-6 Levels Using Enzyme-Linked Immunosorbent Assay

Synovial fibroblast cells from RA patients (Patient No. R41 to No. R45, *n* = 5) were cultured and treated with different concentrations (0, 10, 50, and 100 μM) of UDP (Sigma-Aldrich, Germany), and the supernatants were collected at 24 h. The rat joint cavity lavage fluid was collected as a synovial fluid sample by flushing with 0.5 ml of PBS. The concentration of human or rat IL-6 was measured with an ELISA kit (eBioscience, United States) according to the protocol. In brief, a 100 μL volume of the standard, control or samples was added to each well and incubated for 2 h at room temperature. After three washes, 200 μL of human IL-6 conjugate antibody was added to each well, incubated for 2 h at room temperature and washed three times. A 200 μL aliquot of substrate solution was then added to each well and incubated for 20 min at room temperature. Then, 50 μL of Stop Solution was added to each well, and the optical density of each well was measured at 450 nm in a microplate reader (BioTek, United States).

### Measurement of IL-6 Levels Using Flow Assay

RA synovial fibroblast cells (Patient No. R41 to No. R45, *n* = 5) were isolated and seeded in 96-well plates at a density of 3 × 10^4^ cells per well and incubated overnight. UDP and MRS2578 were dissolved in PBS vehicle containing 0.1% DMSO. The cells were incubated with or without MRS2578 (Med Chem Express, United States) at a final concentration of 10 μM for 1 h. UDP (Sigma-Aldrich, Germany) was then added at a final concentration of 100 μM, and incubation was continued for 24 h. The supernatants were collected after centrifugation at 1,000 × g for 20 min. The IL-6 concentrations in the supernatants were quantified using a human IL-6 flow assay kit (Cell Gene, China). In brief, anti-IL-6 antibodies were conjugated to fluorescence-encoded beads, and the beads and biotinylated anti-IL-6 detection antibodies were mixed with the samples. Streptavidin-PE was added, and the mixture was incubated with shaking for 2 h at room temperature. The beads were washed and then analyzed in a NovoCyte D2040R flow cytometer (ACEA Biosciences, United States). The data were analyzed using FlowJo software (Tree Star, United States).

Rat peripheral blood was collected from rat inferior vena cava, and the IL-6 level was measured using a similar protocol as the rat IL-6 capture bead B6 product commercially obtained from BioLegend. The data were analyzed using LEGENDplex v8.0 software (BioLegend).

### Evaluation of Synovial Fibroblast Cell Proliferation Using a CCK-8 Assay

RA synovial fibroblast cells (Patient No. R41 to No. R45, *n* = 5) were treated with different concentrations (0 μM, 10 μM, 50 μM, or 100 μM) of UDP for 0, 6, 12, and 24 h. A 10 μL volume of CCK-8 solution (Dojindo, Japan) was added to each well and incubated for an additional 4 h. The absorbance was measured at 450 nm in a spectrophotometer (BioTek, United States).

### Evaluation of Synovial Fibroblast Cell Proliferation Using Real-Time Cell Analysis

A dual-plate RTCA instrument (ACEA Biosciences, United States) was placed in a humidified incubator maintained at 37°C and 5% CO_2_. RA synovial fibroblast cells (Patient No. R41 to No. R45, *n* = 5) were isolated and seeded in cell culture E-plates (1 × 10^4^ cells per well) (ACEA Biosciences, United States) and treated with 100 μM UDP with or without MRS2578 (MedChemExpress, United States) at a final concentration of 10 μM for 3 days. The 96-well E-plate was monitored every 30 min for 48 h, and cell proliferation was monitored in real time by measuring the electrical impedance using the xCELLigence RTCA TP System (ACEA Biosciences, United States). The cell growth curves were automatically recorded based on continuous quantitative monitoring of cell proliferation. The data were analyzed with Real-Time Cell Analyzer software (version 1.2).

### Detection of Synovial Fibroblast Cell Apoptosis Using Flow Cytometry

RA synovial fibroblast cells (Patient No. R41 to No. R45, *n* = 5) were cultured and treated with or without MRS2578 at a final concentration of 10 μM for 1 h. Then, UDP at a final concentration of 100 μM was added, and incubation was continued for 24 h. Cells (6 × 10^4^) were collected and resuspended in binding buffer. An Annexin V-FITC-conjugated antibody and a PI-conjugated antibody (BioLegend) were added to the suspended cells. Apoptosis was detected by flow cytometry.

### Cell Migration Assay

RA synovial fibroblast cells (Patient No. R41 to No. R45, *n* = 5) were isolated and seeded in 6-well plates. When the cells were 80–90% confluent, the wound healing assay was conducted by scratching the cell layer in each well with a sterile P200 pipette tip. The cells were preincubated with or without MRS2578 at a final concentration of 10 μM for 1 h. UDP at a final concentration of 100 μm was then added, and incubation was continued for 24 h. The cells were photographed at 0 and 24 h (Olympus IX51, Japan), and the wound area was calculated with ImageJ software (NIH, Bethesda, MD, United States).

### Transwell Assay

RA synovial fibroblast cells (Patient No. R41 to No. R45, *n* = 5) (1 × 10^4^ cells/ml) in serum-free medium were seeded in the upper compartments of Matrigel invasion chambers (Corning, United States). Medium containing 10% FBS was added to the lower compartments of the chambers. The cells were incubated with or without MRS2578 at a final concentration of 10 μM for 1 h. UDP (Sigma-Aldrich, Germany) at a final concentration of 100 μM was then added, and incubation was continued for 24 h. The cells on the top surface of the membrane were removed with cotton swabs, and the cells that penetrated to the bottom surface of the membrane were stained with crystal violet. Images were acquired by fluorescence microscopy (Olympus IX51, Japan), and the cells were quantified with ImageJ software (NIH, Bethesda, MD, United States).

### Sources of Microarray Data

The expression level of P2Y6 in RA or OA synovial tissues was analyzed in four published gene expression profile datasets (dataset type: expression profiling by array) in the Gene Expression Omnibus (GEO, https://www.ncbi.nlm.nih.gov/geoprofiles) database. The expression data of 10 patients with RA and six OA controls from dataset GDS5402/208,373_s_at, five patients with RA and five OA controls from dataset GDS2126/38222_at, 10 patients with RA and 10 OA controls from dataset GDS5401/208373_s_at, and 13 patients with RA and 10 OA controls from dataset GDS5403/208373_s_at were analyzed in SPSS software v. 21.0 (IBM, United States) using an unpaired Student’s t-test.

### RNA Isolation and Quantitative Real-Time PCR

Human (Patient No. R41 to No. R50, *n* = 10) and rat synovial tissues were collected as described above. Total RNA was isolated using TRIzol reagent (Invitrogen) and reverse transcribed to cDNA (Vazyme, China). Reak-time PCR was performed in a StepOnePlus™ Real-Time PCR System (Thermo Fisher Scientific, United States) using iQ SYBR Green Supermix (Bio-Rad) according to the manufacturer’s guidelines. The PCR primers were designed as follows: human P2Y6 sense: 5′-GTG​TCT​ACC​GCG​AGA​ACT​TCA-3′, human P2Y6 antisense: 5′-CCA​GAG​CAA​GGT​TTA​GGG​TGT​A-3′; human *β*-actin sense: 5′-CAT​GTA​CGT​TGC​TAT​CCA​GGC-3′, human *β*-actin antisense: 5′-CTC​CTT​AAT​GTC​ACG​CAC​GAT-3′; rat P2Y6 sense: 5′-GTG​GTA​TGT​GGA​GTC​GTT​TGA-3′, rat P2Y6 antisense: 5′-CTG​TAG​GAG​ATC​GTG​GTT-3′; rat GAPDH sense: 5′-TCC​CTC​AAG​ATT​GTC​AGC​AA-3′, rat GAPDH antisense: 5′-AGA​TCC​ACA​ACG​GAT​ACA​TT-3′. The PCR primers were designed based on a study by Kim (Kim et al., 2011).

### Evaluation of P2Y6 Expression Using Western Blotting

Human synovial tissues (Patient No. R46 to No. R50, *n* = 5) were collected as described above. Samples were homogenized on ice in radioimmunoprecipitation assay (RIPA) lysis buffer (Beyotime), separated by 12% sodium dodecyl sulfate-polyacrylamide gel electrophoresis (SDS-PAGE) and transferred to polyvinylidene fluoride (PVDF) membranes (Millipore, United States). Membranes were incubated with a rabbit anti-P2Y6 antibody (Abcam, Catalog number: ab198805) and horseradish peroxidase (HRP)-conjugated goat anti-rabbit IgG (Abcam, Catalog number: ab205718). *β*-Actin (Abcam, Catalog number: ab115777) was used as the internal reference for normalization of the P2Y6 expression level. Immunoreactive bands were visualized using Western Chemiluminescent Horseradish Peroxidase Substrate (ECL, Millipore), and band densities were quantified using ImageJ software (NIH, Bethesda, MD, United States).

### Evaluation of P2Y6 Expression Using Immunofluorescence

Paraffin sections of human synovial tissues (Patient No. R46 to No. R50, *n* = 5) were permeabilized with 0.05% Triton X-100 for 10 min, blocked with 5% goat serum for 1 h, and incubated with a rabbit anti-P2Y6 antibody (1:200, Abcam, Catalog number: ab198805) at 4°C overnight. The tissue sections were incubated with goat anti-rabbit IgG H&L (Alexa Fluor^®^ 555) (1:200, Abcam, ab150078) for 1 h in the dark. Nuclei were stained with 4ʹ,6-diamidino-2-phenylindole (DAPI) (Abcam, ab228549). Images were acquired under a fluorescence microscope, and quantification of the signal density was conducted in Image-Pro Plus 6.0 (Media Cybernetics, Inc., Rockville, MD, United States).

### Examination of P2Y6 Expression Using Immunohistochemical Staining

Paraffin sections of human synovial tissues (Patient No. R46 to No. R50, *n* = 5) were incubated first with a rabbit anti-P2Y6 antibody (1:200, Abcam, Catalog number: ab198805) at 4°C overnight and then with HRP-conjugated goat anti-rabbit IgG (Abcam, Catalog number: ab205718). Sections were treated with diaminobenzidine (DAB) and counterstained with hematoxylin. The results were analyzed, and the expression level was quantified in ImageJ software (NIH, Bethesda, MD, United States).

### Statistical Analysis

Statistical analyses were performed using GraphPad Prism 7.0 (GraphPad, United States) and SPSS software v.21.0 (IBM, United States). The significance of differences between groups was evaluated using Student’s unpaired *t*-test. Differences with *p* values of <0.05 were considered significant.

## Results

### Metabolomic Analysis of RA Synovial Fluids

**FIGURE 1:**
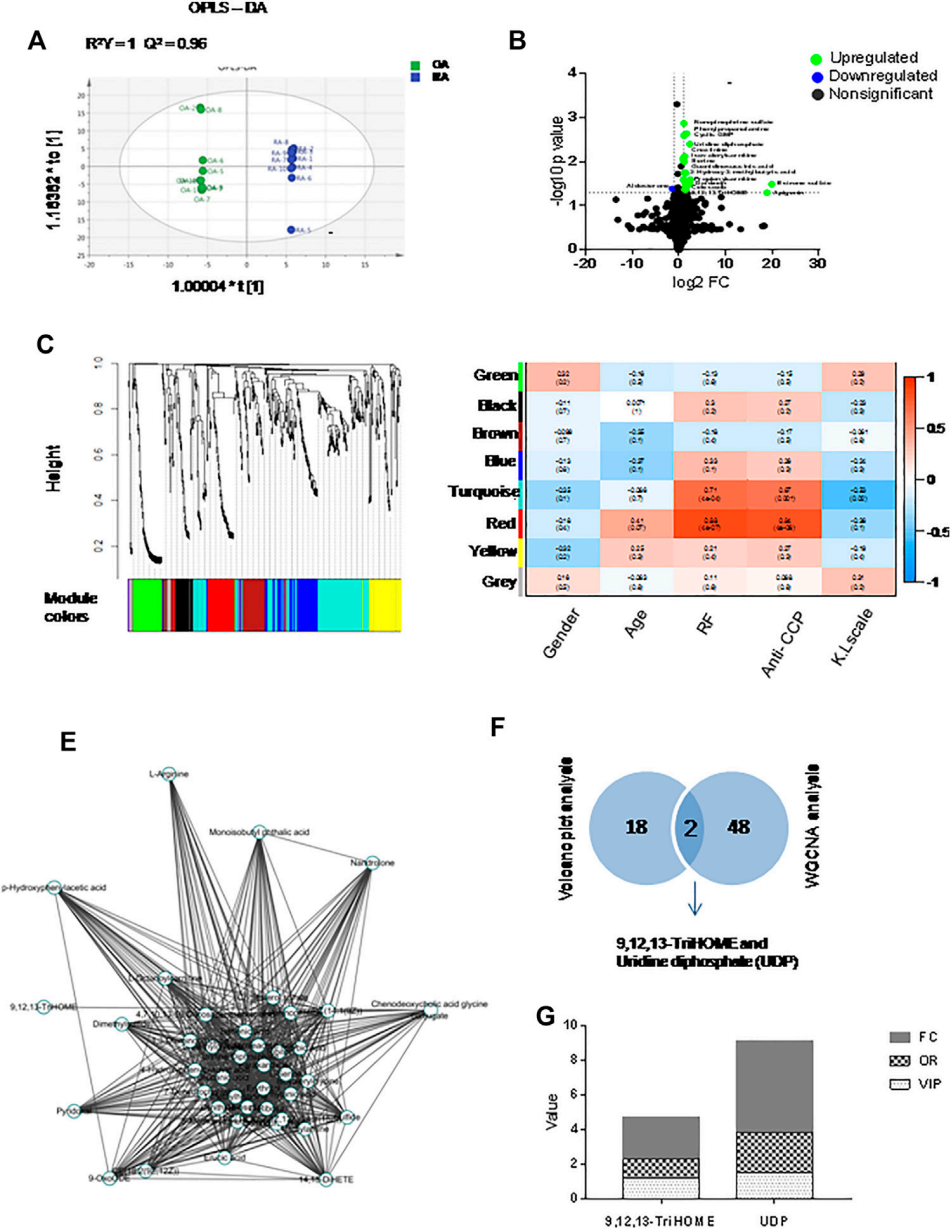
Multivariate statistical analysis for metabolomics data with synovial fluids. **(A)** OPLS-DA of synovial fluids (*n* = 10 for RA and OA). **(B)** Volcano plots indicating the statistical significance of metabolite changes between RA and OA samples (*p* < 0.05; FC > 2). **(C)** Clustering dendrograms of the metabolite sets. **(D)** Correlation of metabolite coexpression network modules with the clinical features of RA patients. **(E)** Metabolite expression network derived from the red module. **(F)** Venn diagrams of key metabolites in the volcano plot analysis and WGCNA sets. **(G)** Histogram of key metabolites (9,12,13-TriHOME and UDP) based on FC, OR, and VIP.

Samples of RA (*n* = 10) and OA (*n* = 10) synovial fluids were analyzed using an LC-MS approach. A total of 481 variables were identified after searching the Human Metabolome Database (HMDB). Orthogonal partial least squares-discriminant analysis (OPLS-DA), the most frequently used multivariate statistical method, was applied for metabolomic analysis. The R^2^Y of the OPLS-DA model was 1, and the Q^2^ was 0.96, indicating that the model was stable and reliable. The RA and OA OPLS-DA score plots were separated, indicating that the model could discriminate metabolites between RA and OA (Figure 1A). Volcano plots were constructed by analyzing the fold change (FC), *p* values from the *t*-test results and the variable importance in projection (VIP) scores from OPLS-DA. Metabolites meeting the criteria of FC > 2 or <0.5, *p* < 0.05 and VIP>1 were defined as differentially expressed metabolite DEMs. The concentrations of the following metabolites were significantly elevated in RA synovial fluids compared with the OA samples (Figure 1B). The above data are provided in Supplementary Table S3. Using the metabolite sets, eight variable network modules including green (*n* = 51), black (*n* = 31), brown (*n* = 56), blue (*n* = 66), turquoise (*n* = 144), red (*n* = 50), yellow (*n* = 54) and gray (*n* = 27) via WGCNA were identified. Each leaf in the tree represents one metabolite (Figure 1C). Correlations between these metabolite network modules and clinical prognostic data [including sex, age and RF level, anti-CCP level and Kellgren-Lawrence (K&L) score] were analyzed. The turquoise module was positively correlated with RF (r = 0.71, *p* = 0.0004) and anti-CCP levels (r = 0.67, *p* = 0.001). The red module was positively correlated with RF (r = 0.88, *p* < 0.0001) and anti-CCP levels (r = 0.84, *p* < 0.0001) (Figure 1D). Cytoscape 3.6.1 was used to visualize the metabolite network in the red module. The results are shown in Figure 1E. Two metabolites (UDP and 9,12,13-TriHOME) were found in both the red module identified by WGCNA and the DEMs identified by volcano plot analysis (Figure 1F), indicating that they might play an important role in the pathological progression of RA. By comprehensive analysis of the VIP scores (VIP = 1.54), odds ratios (ORs) in logistic regression (OR = 2.314), FC values (FC = 5.2) and *p* value (*p* = 0.0039), UDP was considered to be very important for RA, and we decided to concentrate on the pathogenic role of UDP in the subsequent study (Figure 1G). The above data are also provided in Supplementary Table S3.

### UDP Level in RA Peripheral Blood and Synovial Fluids

**FIGURE 2:**
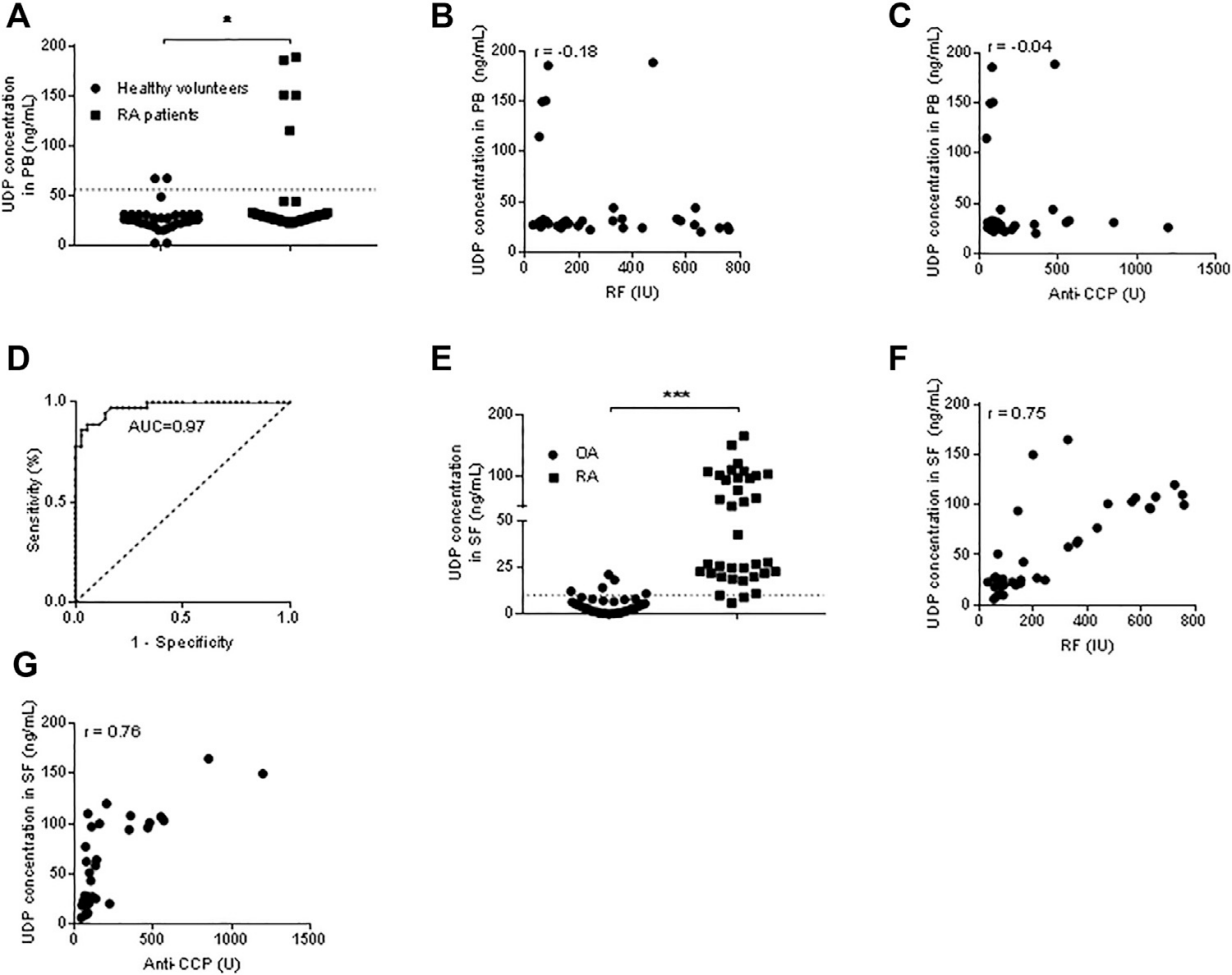
UDP levels in blood and synovial fluids. **(A)** The UDP level in peripheral blood (*n* = 36 for RA patients and healthy volunteers) was measured using a Transcreener UDP assay. **(B)** Association analysis of the RA blood UDP level with the serum RF level. **(C)** Association analysis of the RA blood UDP level with the serum anti-CCP level. **(D)** ROC analysis of UDP in RA synovial fluids. **(E)** The UDP level in synovial fluids (*n* = 36 for RA and OA) was measured using a Transcreener UDP assay. **(F)** Association analysis of the RA synovial fluid UDP level with the serum RF level. **(G)** Association analysis of the RA synovial fluid UDP level with the serum anti-CCP level. PB: peripheral blood, SF: synovial fluids. *p < 0.05, **p < 0.01 and ***p < 0.001.

The UDP concentration was measured in the plasma of RA patients (*n* = 36) and healthy volunteers (*n* = 36) using the Transcreener UDP Assay. Compared with those in the blood of healthy volunteers, the UDP levels in the blood of patients with RA were significantly increased (Figure 2A). Pearson correlation analysis was performed, and no correlation was detected between the blood UDP level and serum RF level (R = -0.18) (Figure 2B) or between the UDP level and serum anti-CCP level (R = -0.04) (Figure 2C). ROC analysis was used to examine possible associations between blood UDP levels and RF and anti-CCP levels in RA patients. Normally, an area under the ROC curve (AUC) of >0.9 was considered excellent, 0.8–0.9 was considered very good, 0.7–0.8 was considered good, 0.6–0.7 was considered average, and <0.6 was considered poor. The AUC value of UDP in the present experiment was 0.97, which indicated an excellent diagnostic test for RA (Figure 2D). The UDP concentration was also measured in RA synovial fluids (*n* = 36) and OA samples (*n* = 36). Compared with that in OA synovial fluids, the UDP level in RA synovial fluids was significantly increased (Figure 2E). Pearson correlation analysis detected a moderate positive correlation (R = 0.75) between the synovial fluid UDP level and serum RF level (Figure 2F) and between the synovial fluid UDP level and serum anti-CCP level (R = 0.76) (Figure 2G).

We further grouped the patients based on their genders and measured their UDP levels. In peripheral blood, the UDP level was significantly increased in male RA patients (*n* = 5) compared with healthy male volunteers (*n* = 15). There was no significant difference in UDP levels between female RA patients (*n* = 31) and female healthy volunteers (*n* = 21). Additionally, the UDP level was significantly higher in healthy females than in healthy males. In synovial fluids, the UDP level was significantly increased in both male (*n* = 5) and female (*n* = 31) RA patients compared with OA male (*n* = 17) and female counterparts (*n* = 19). There was no significant difference in UDP levels between female RA patients and male RA patients or between male OA patients and female OA patients. The results are shown in Supplementary Figure S1. Thus, the UDP level was significantly elevated in synovial fluid samples from RA patients, regardless of whether the samples were collected from male patients or female patients. SF UDP level in ACPA/RF-positive-positive RA was significantly higher than that in ACPA/RF-negative RA (Supplementary Figure S2).

### The Effect of UDP on RA Synovial Fibroblast Cells

**FIGURE 3:**
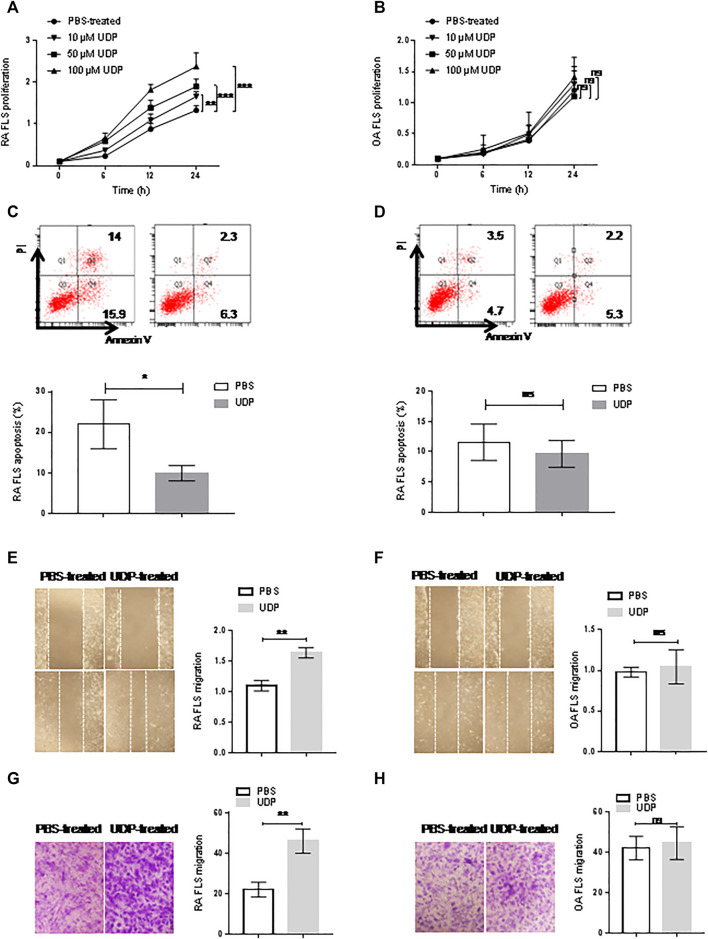
The effect of UDP on synovial fibroblast cells. Cell proliferation of RA **(A)** or OA **(B)** synovial fibroblast cells (*n* = 5 for each disease) was assessed using a CCK-8 assay. Apoptosis of RA **(C)** or OA **(D)** synovial fibroblast cells (*n* = 5 for each disease) was assessed using flow cytometry and statistical analysis. Cell migration of RA **(E)** or OA **(F)** synovial fibroblast cells (*n* = 5 for each disease) was assessed using a wound healing assay and statistical analysis. **(G)** Cell migration of RA or OA **(H)** synovial fibroblast cells (*n* = 5 for each disease) was assessed using a Transwell assay and statistical analysis. These experiments were repeated three times. FLS: synovial fibroblast cells. *p < 0.05, **p < 0.01 and ***p < 0.001.

Cultured synovial fibroblast cells from RA patients (*n* = 5) or OA patients (*n* = 5) were treated with different concentrations of UDP. The CCK-8 assay showed increased cell proliferation of RA synovial fibroblast cells in the presence of 10, 50, and 100 μM UDP compared with that in the PBS-treated controls (Figure 3A). However, this assay did not show a significant change in the proliferation of OA synovial fibroblast cells in the presence of 10 μM, 50 μM or 100 μM UDP compared with that in the PBS-treated controls (Figure 3B). Annexin V/PI apoptosis analysis showed that the apoptosis rate of RA synovial fibroblast cells was decreased in the presence of UDP (100 μM) compared with that in the PBS-treated controls (Figure 3C). However, this analysis detected little change in OA synovial fibroblast cell apoptosis in the presence of UDP compared with that in the PBS-treated controls (Figure 3D).

Wound healing and Transwell assays were used to evaluate the effect of UDP on the migration of RA synovial fibroblast cells. The wound healing assays showed that RA synovial fibroblast cell migration was increased in the presence of UDP (100 μM) compared with that of PBS-treated controls (Figure 3E). The Transwell assays also showed that RA synovial fibroblast cell migration was significantly increased in the presence of UDP (100 μM) compared with that of PBS-treated controls (Figure 3F). However, the wound healing assay showed only a slight change in OA synovial fibroblast cell migration in the presence of UDP (100 μM) compared with that of PBS-treated controls (Figure 3G). The Transwell assay also showed only a slight change in OA synovial fibroblast cell migration in the presence of UDP (100 μM) compared with that of PBS-treated controls (Figure 3H).

**FIGURE 4:**
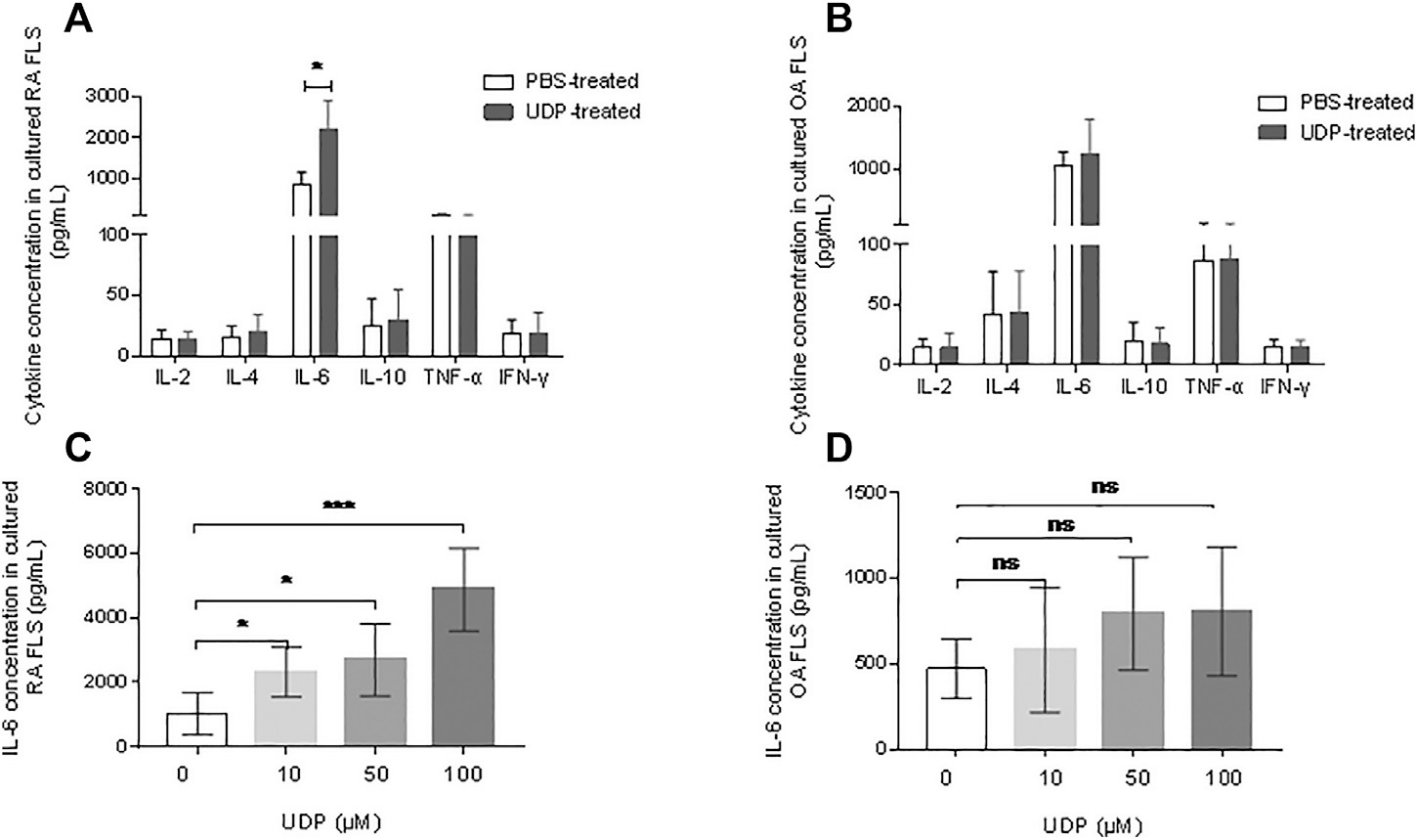
The effect of UDP on cytokine secretion of synovial fibroblast cells. Cytokine secretion in cultured medium of RA **(A)** or OA **(B)** synovial fibroblast cells (*n* = 5 for each disease) was assessed using a flow cytometric bead assay. IL-6 secretion in the culture medium of RA **(C)** or **(D)** synovial fibroblast cells (*n* = 5 for each disease) was also assessed using ELISA. These experiments were repeated three times. FLS: synovial fibroblast cells. *p < 0.05, **p < 0.01 and ***p < 0.001.

Flow cytometry was used to examine proinflammatory cytokine levels in cultures of synovial fibroblast cells. The assay showed significantly elevated IL-6 levels in the culture medium of RA synovial fibroblast cells in the presence of 100 μM UDP, but the concentrations of IL-2, IL-4, IL-10, TNF-α and IFN-γ were not significantly changed compared with those in the PBS-treated controls (Figure 4A). Compared with those in the control cultures, the respective concentrations of IL-2, IL-4, IL-6, IL-10, TNF-α and IFN-γ in the culture medium of OA synovial fibroblast cells were not significantly changed in the presence of 100 μM UDP **(** Figure 4B **)**.

An IL-6 ELISA was performed to verify the above results. This assay showed significantly increased IL-6 levels in the culture medium of RA synovial fibroblast cells in the presence of 10 μM, 50 μM, and 100 μM UDP compared with that in samples from PBS-treated controls (Figure 4C). However, ELISA showed only slight changes in IL-6 levels in the culture medium of OA synovial fibroblast cells in the presence of 10 μM, 50μM, and 100 μM UDP (Figure 4D). The above results indicated that UDP activated RA synovial fibroblast cells and induced IL-6 secretion rather than other proinflammatory cytokines.

### Regulation of P2Y6 Expression on the Effects of UDP

**FIGURE 5:**
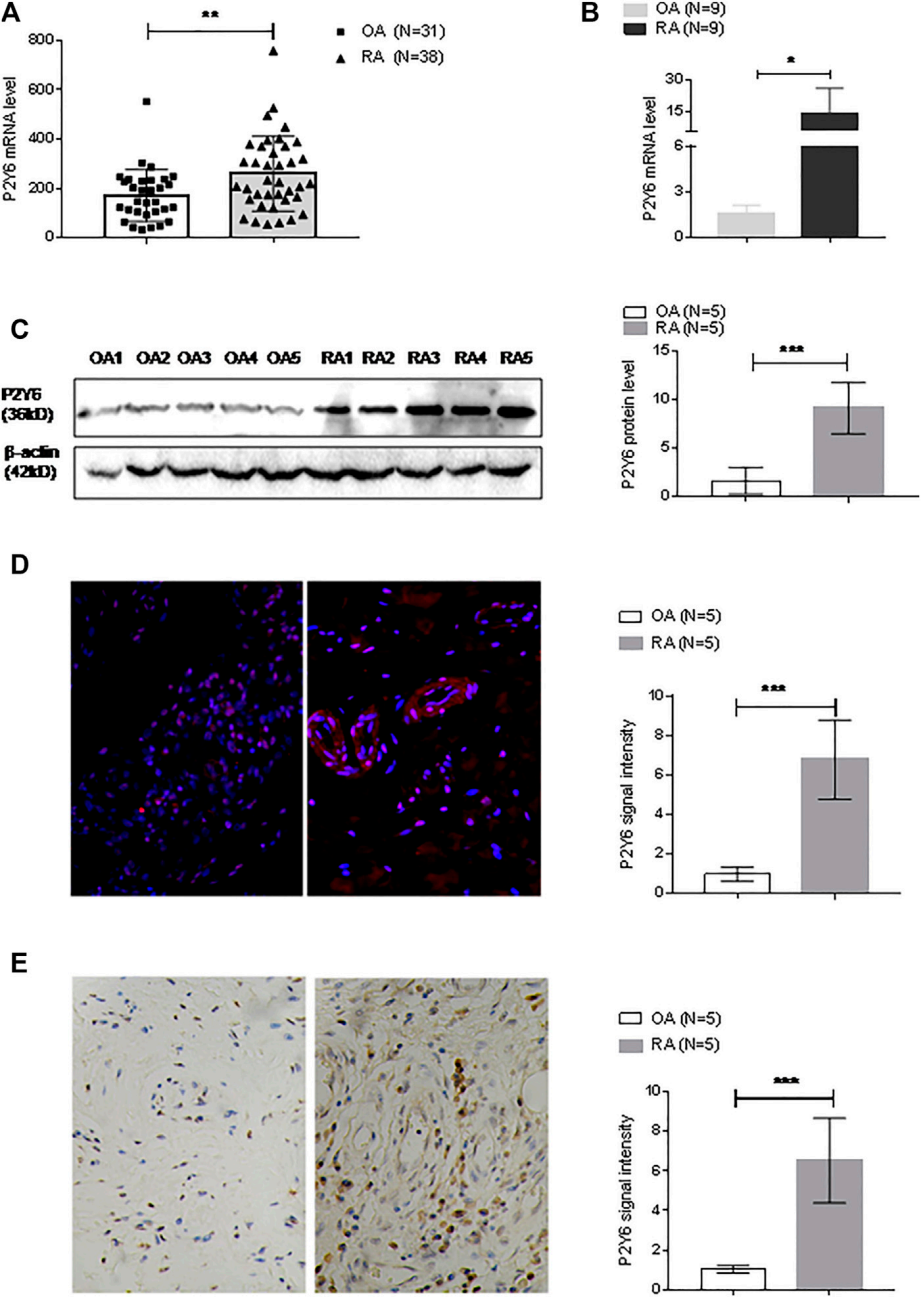
P2Y6 expression in synovial tissues and cultured synovial fibroblast cells. **(A)** P2Y6 expression levels were analyzed based on the GEO database based on synovial tissue microarray data of RA synovial tissue (*n* = 38) and OA (*n* = 31). **(B)** P2Y6 mRNA expression in synovial tissues from RA or OA patients (*n* = 9 for each disease) was assessed using real-time PCR. **(C)** P2Y6 protein expression in synovial tissues from RA or OA patients (*n* = 5 for each disease) was assessed using Western blotting and statistical analysis. **(D)** P2Y6 protein expression in RA or OA synovial tissues (*n* = 5 for each disease) was assessed using immunofluorescence and statistical analysis. **(E)** P2Y6 expression in RA or OA synovial tissues (*n* = 5 for each disease) was assessed using immunohistochemical staining and statistical analysis. These experiments were repeated three times. *p < 0.05, **p < 0.01 and ***p < 0.001.

The abundance of P2Y6 mRNA in RA and OA synovial tissue was analyzed with data from the GEO database. The expression datasets were obtained from expression profiling by arrays for 38 RA synovial tissues and 31 OA synovial tissues. Significantly increased transcription of P2Y6 was found in RA synovial tissues compared with OA samples (Figure 5A).

The expression level of P2Y6 was also examined in synovial tissues that we collected. Real-time PCR detected significantly increased P2Y6 mRNA expression in RA synovial tissues (*n* = 9) compared with OA synovial tissues (*n* = 9) (Figure 5B). Western blot analysis also detected significantly increased expression of P2Y6 protein in RA synovial tissues (*n* = 5) compared with OA synovial tissues (*n* = 5) (Figure 5C). Immunofluorescence analysis detected P2Y6 protein expression in RA synovial tissues (*n* = 5), and the signal density was much higher in the RA samples than in OA synovial tissues (*n* = 5) (Figure 5D). Immunohistochemical analysis detected significantly increased P2Y6 protein expression in RA synovial tissues (*n* = 5) compared with OA synovial tissues (*n* = 5) (Figure 5E).

**FIGURE 6:**
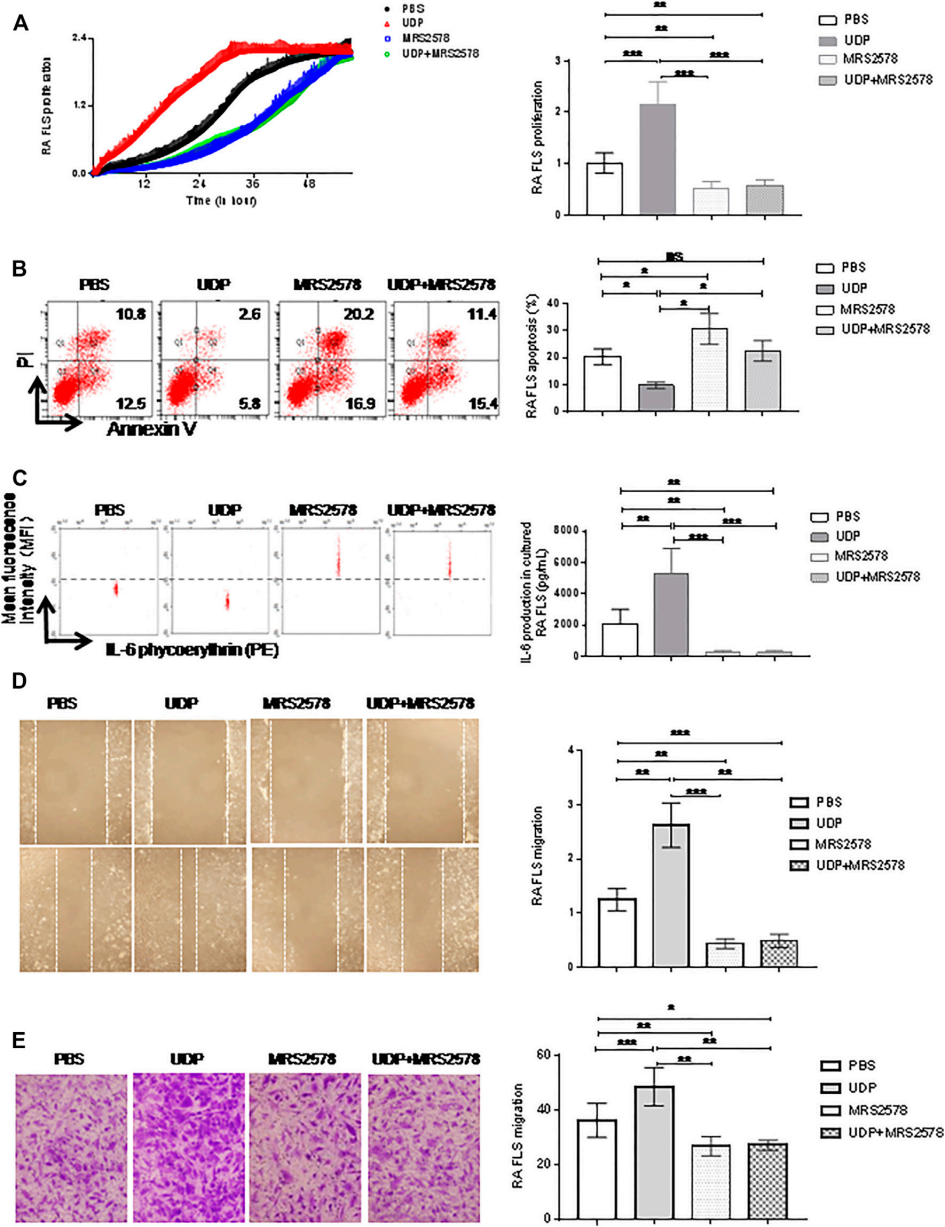
The Effect of MRS2578 on cell activities and IL-6 production in cultured synovial fibroblast cells. The cultured synovial fibroblast cells of RA (*n* = 5) were treated with UDP (100 μM) and/or MRS2578 (10 μM). **(A)** Cell proliferation was assessed using RTCA and statistical analysis. **(B)** Apoptosis was assessed using flow cytometry and statistical analysis. **(C)** IL-6 secretion was assessed using a flow cytometric bead assay and statistical analysis. **(D)** Cell migration was assessed using a wound healing assay and statistical analysis. **(E)** Cell migration was assessed using a Transwell assay and statistical analysis. These experiments were repeated three times. * FLS: synovial fibroblast cells. *p < 0.05, **p < 0.01 and ***p < 0.001.

To determine the effect of P2Y6 on RA synovial fibroblast cells, RA synovial fibroblast cells (*n* = 5) were cultured with both UDP (100 μM) and MRS2578 (10 μM). Cell proliferation was investigated using RTCA, apoptosis was detected with an annexin V/PI apoptosis assay, and IL-6 secretion was measured using a flow cytometric bead assay. Compared with that of PBS-treated controls, synovial fibroblast cell proliferation was increased in the presence of UDP and was decreased in the presence of MRS2578 alone or in the presence of both UDP and MRS2578 (Figure 6A). Compared with that in the PBS-treated culture, RA synovial fibroblast cell apoptosis was decreased in the presence of UDP alone, increased in the presence of MRS2578 alone, and unchanged in the presence of UDP and MRS2578 (Figure 6B). Compared with that in the PBS-treated culture, IL-6 secretion in RA synovial fibroblast cells was increased in the presence of UDP and was decreased in the presence of MRS2578 alone and in the presence of both UDP and MRS2578 (Figure 6C). The wound healing assay showed that RA synovial fibroblast cell migration was increased in the presence of UDP and was decreased in the presence of MRS2578 alone and in the presence of UDP and MRS2578 together compared with that in the PBS-treated controls (Figure 6D). Moreover, the Transwell assay also showed that RA synovial fibroblast cell migration was increased in the presence of UDP and was decreased in the presence of MRS2578 and in the presence of UDP and MRS2578 together compared with that in the PBS-treated controls (Figure 6E). The above results demonstrated that MRS2578 suppressed the pathogenic activities of RA synovial fibroblast cells.

### The Effect of UDP on CIA

**FIGURE 7:**
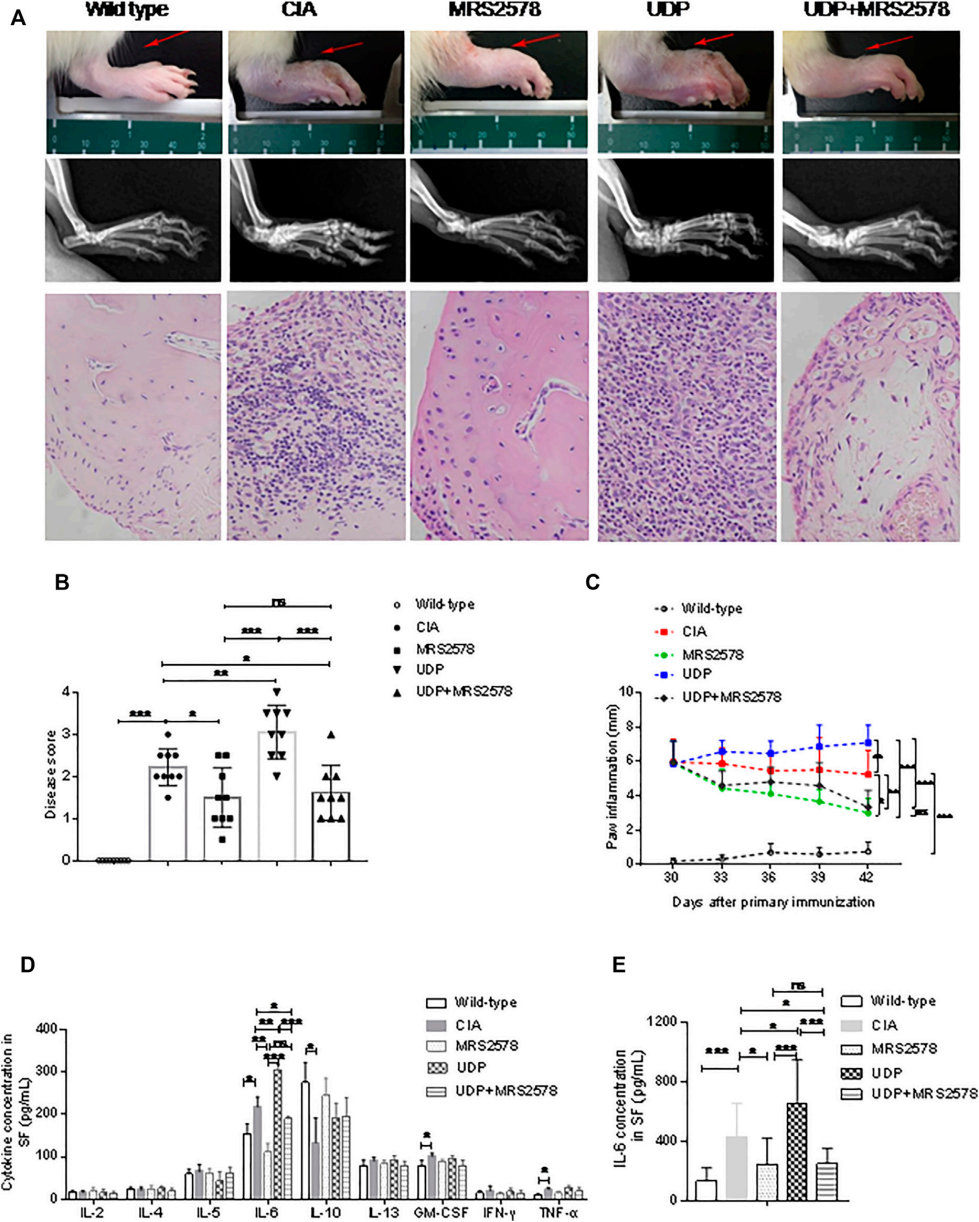
The effect of MRS2578 on CIA rats treated with UDP. **(A)** X-ray images and histochemical staining images of joint inflammation. **(B)** Disease scores were quantified based on histologic evidence. **(C)** Inflammation curve analysis based on paw inflammation. **(D)** Cytokine levels in synovial fluids using a flow cytometric bead assay. **(E)** IL-6 levels in synovial fluids were assessed using ELISA. X-ray images, joint tissues, peripheral blood and synovial fluids were collected 20 days after the first UDP injection (3 days after the last UDP injection/7 weeks after the first collagen injection). Each groups had nine rats, SF: synovial fluids. *p < 0.05, **p < 0.01 and ***p < 0.001.

Rats with CIA were simultaneously injected with UDP, MRS2578 or both UDP and MRS2578. The disease activity as assessed by toe swelling was increased, and radiological signs (soft tissue swelling, new bone formation and marginal osseointegration) and histochemical staining were significantly enhanced in the CIA rats compared with the normal control rats, indicating successful establishment of CIA in the rats. Compared with CIA rats, the disease score was significantly increased in CIA rats treated with UDP but decreased in CIA rats treated with MRS2578 or with both UDP and MRS2578. Compared with CIA rats treated with UDP, the disease score was decreased in CIA rats treated with MRS2578 or with both UDP and MRS2578. The disease score did not differ significantly between the CIA rats treated with MRS2578 alone and the CIA rats treated with both UDP and MRS2578 (Figures 7A,B). Compared with that in normal control rats, paw inflammation in CIA rats was progressively exacerbated, indicating successful establishment of CIA in the rats. Compared with CIA rats, paw inflammation was significantly exacerbated in CIA rats treated with UDP but alleviated in CIA rats treated with MRS2578 or with both UDP and MRS2578. Compared with CIA rats treated with UDP, paw inflammation was alleviated in CIA rats treated with MRS2578 or with both UDP and MRS2578. Paw inflammation did not significantly differ between CIA rats treated with MRS2578 alone and CIA rats treated with both UDP and MRS2578 (Figure 7C).

Synovial fluids were collected from rats on day 20 after the first UDP injection, and the cytokine concentrations were measured by flow cytometry. Compared with the corresponding concentrations in the normal control group, the concentrations of IL-6, GM-CSF and TNF-α in the CIA control group were significantly increased, the concentrations of IL-10 were significantly decreased, and the concentrations of IL-2, IL-4, IL-5, IL-13, and IFN-γ were not significantly changed. The increased IL-6, GM-CSF and TNF-α levels and decreased IL-10 levels indicated successful establishment of CIA in the rats. Compared with that in CIA rats, the IL-6 level was significantly increased in CIA rats treated with UDP but decreased in CIA rats treated with MRS2578 or with both UDP and MRS2578. Compared with that in CIA rats treated with UDP, the IL-6 level was decreased in CIA rats treated with MRS2578 or with both UDP and MRS2578. However, the IL-6 level did not differ significantly between CIA rats treated with MRS2578 alone and CIA rats treated with both UDP and MRS2578. Moreover, the concentrations of IL-2, IL-4, IL-5, IL-10, IL-13, GM-CSF, IFN-γ and TNF-α did not differ significantly between UDP-treated and MRS2578-treated CIA rats or between UDP-treated rats and CIA rats treated with both UDP and MRS2578 (Figure 7D). Cytokine expression in synovial fluids of rats was verified by IL-6 ELISA. The IL-6 level was significantly increased in CIA rats treated with UDP but decreased in CIA rats treated with MRS2578 or with both UDP and MRS2578 compared with CIA rats. Compared with that in CIA rats treated with UDP, the IL-6 level was decreased in CIA rats treated with MRS2578 or with both UDP and MRS2578. However, the IL-6 level did not differ significantly between CIA rats treated with MRS2578 alone and CIA rats treated with both UDP and MRS2578 (Figure 7E).

**FIGURE 8:**
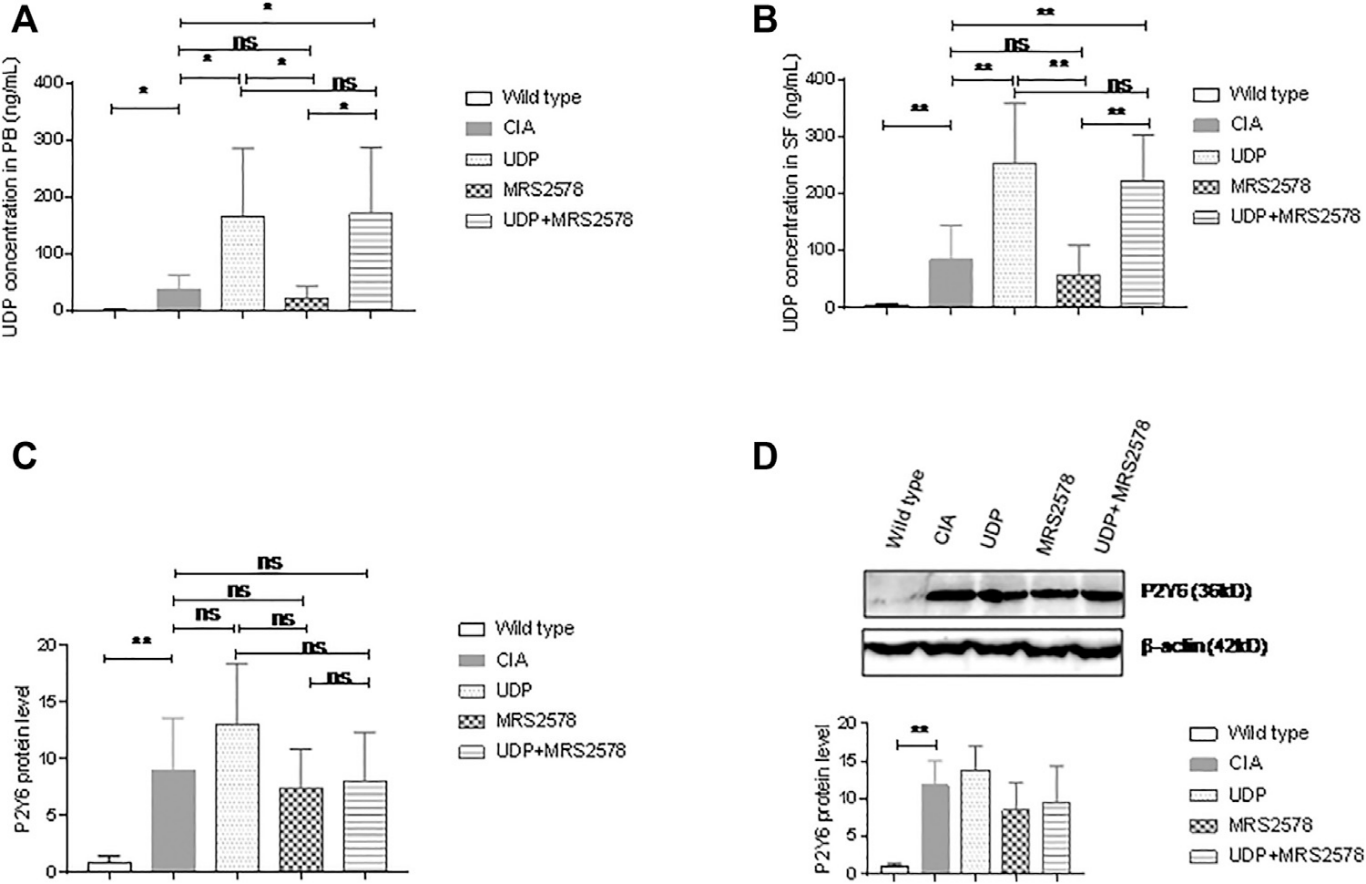
UDP levels in synovial fluids and P2Y6 expression in synovial tissues of CIA rats. **(A)** The UDP levels in rat peripheral blood were assessed using fluorescence polarization analysis. **(B)** The UDP levels in rat synovial fluids were assessed using fluorescence polarization analysis. **(C)** P2Y6 expression in rat synovial tissues was assessed using real-time PCR. **(D)** P2Y6 expression in synovial tissues was assessed using Western blotting and statistical analysis. Each groups had nine rat samples. PB: peripheral blood, SF: synovial fluids. *p < 0.05, **p < 0.01 and ***p < 0.001.

We examined UDP levels in the peripheral blood and synovial fluids of CIA rats using the fluorescence polarization method. The UDP level in peripheral blood was significantly higher in CIA rats than in normal control rats. Compared with the UDP level in CIA rats, the peripheral blood UDP level was significantly increased in CIA rats treated with UDP or both UDP and MRS2578 but was not significantly different in CIA rats treated with MRS2578. Compared with that in CIA rats treated with UDP, the peripheral blood UDP level was decreased in CIA rats treated with MRS2578 but was not significantly different in CIA rats treated with both UDP and MRS2578. The UDP level in peripheral blood was significantly higher in CIA rats treated with both UDP and MRS2578 than in CIA rats treated with MRS2578 (Figure 8A). The UDP level in the synovial fluids was significantly higher in CIA rats than in normal control rats. Compared with the UDP level in CIA rats, the UDP level in the fluid samples was significantly increased in CIA rats treated with UDP or both UDP and MRS2578 but was not significantly different in CIA rats treated with MRS2578. Compared with that in CIA rats treated with UDP, the UDP level in the fluid samples was decreased in CIA rats treated with MRS2578 but was not significantly different in CIA rats treated with both UDP and MRS2578. Finally, the UDP level in the fluid samples was significantly higher in CIA rats treated with both UDP and MRS2578 than in CIA rats treated with MRS2578 alone (Figure 8B).

We also examined P2Y6 expression in synovial tissues of the model rats by real-time PCR and Western blot analysis. P2Y6 mRNA expression in synovial samples was significantly higher in CIA rats than in normal control rats. P2Y6 mRNA expression in synovial tissues did not differ significantly between CIA rats and CIA rats treated with UDP alone, between CIA rats and CIA rats treated with MRS2578 alone or between CIA rats and CIA rats treated with UDP and MRS2578 together. P2Y6 mRNA expression in synovial tissues did not differ significantly between UDP-treated CIA rats and MRS2578-treated CIA rats or between UDP-treated rats and both UDP- and MRS2578-treated rats. P2Y6 mRNA expression in synovial tissues did not differ significantly between MRS2578-treated CIA rats and both UDP- and MRS2578-treated CIA rats (Figure 8C). Meanwhile, P2Y6 protein expression in synovial tissues was significantly higher in CIA rats than in normal control rats. However, P2Y6 protein expression in the synovial tissues did not differ significantly between CIA rats and UDP-treated CIA rats, between CIA rats and MRS2578-treated CIA rats or between CIA rats and both UDP- and MRS2578-treated CIA rats. Moreover, P2Y6 protein expression in synovial tissues did not differ significantly between UDP-treated CIA rats and MRS2578-treated CIA rats or between UDP-treated CIA rats and both UDP- and MRS2578-treated CIA rats. P2Y6 protein expression in synovial tissues also did not differ significantly between MRS2578-treated CIA rats and UDP- and MRS2578-treated CIA rats (Figure 8D). The above measurements indicated that UDP or MRS2578 treatment did not change P2Y6 expression in CIA rats, although P2Y6 expression was increased in CIA rats following collagen treatment.

## Discussion

In this study, via metabolomic analysis, we found significantly increased UDP levels in RA synovial fluids compared with OA synovial fluids. We confirmed this finding in blood and synovial fluids by comparing samples from 36 RA patients and 36 OA patients as well as 36 healthy people using the Transcreener UDP Assay. Furthermore, we detected increased levels of UDP in blood and synovial fluids from CIA rats compared with normal control rats. These results suggest a high level of UDP in RA and CIA. Furthermore, the UDP level was moderately correlated with the levels of anti-CCP and RF, indicating the potential role of UDP in RA. There was no significant difference in UDP levels between female RA patients and male RA patients or between male OA patients and female OA patients.

We continued by investigating the effect of a high UDP level on RA and CIA. UDP injection significantly aggravated paw inflammation in CIA rats. Additionally, UDP stimulated the proliferation and migration of RA synovial fibroblast cells *in vitro* and suppressed their apoptosis, indicating the activating effects of UDP on RA synovial fibroblast cells. UDP also increased IL-6 secretion in cultured RA synovial fibroblast cells and in CIA rats but did not affect the production of other cytokines, such as IL-2, IL-4, IL-10, TNF-α and IFN-γ. IL-6 plays a key role in local and systemic manifestations of RA (Abdel Mequid et al., 2013). Blockade of IL-6 has been suggested to be an effective method for RA treatment (Md Yusof and Emergy, 2013; Narazaki et al., 2017). The above results suggest that a high UDP level stimulates the pathogenic progression of RA. This study is the first to report the stimulation of a high UDP level on RA, although some studies have reported that UDP activates inflammatory responses such as phagocytosis and cytokine/chemokine production (Cox et al., 2005; Grbic et al., 2008; Kim et al., 2011).

UDP was described as a ligand for P2Y14 (von Kügelgen and Hoffmann, 2015), but its function is controversial (Fricks et al., 2008; Carter et al., 2009). UDP also plays a role via P2Y6. The human P2Y6 receptor (hP2Y6) is a member of the G protein-coupled pyrimidinergic P2 receptor family that responds specifically to the extracellular nucleotide UDP. P2Y6 is expressed in neutrophils, macrophages, dendritic cells, eosinophils, B cells and T cells and plays roles in apoptosis and cell differentiation, maturation and migration (Le Duc et al., 2017). In our study, we detected high P2Y6 expression in RA synovial tissues using real-time PCR, Western blotting and immunohistochemistry. We also found increased expression of P2Y6 in CIA synovial tissues. MRS2578 was designed and selected to specifically inhibit P2Y6 activity (Mamedova et al., 2004; Neher et al., 2014). Many studies have demonstrated specific inhibition of MRS2578 on P2Y6 activities and used MRS2578 to specifically inhibit P2Y6 activity to block UDP function (Qin et al., 2016; Nakano et al., 2017). When RA synovial fibroblast cells were cultured with both UDP and MRS2578, a P2Y6 antagonist, their proliferation and IL-6 secretion were significantly suppressed, and the apoptosis rate was increased. CIA rats injected with MRS2578 or with both UDP and MRS2578 showed decreased paw inflammation and IL-6 production. P2Y6 expression was relatively low in OA synovial tissues, and UDP had little effect on OA FLS proliferation and IL-6 secretion. These observations suggest that UDP plays a stimulatory role in RA by regulatingP2Y6 activity. Increased UDP levels and high P2Y6 expression stimulate RA and CIA progression. Targeting P2Y6 receptors might be useful for the treatment of RA. However, UDP did not change P2Y6 expression in the present study; P2Y6 protein expression in synovial tissues was not significantly changed in CIA rats treated with UDP alone, MRS2578 alone or UDP and MRS2578 together. The reason that P2Y6 expression is elevated in RA and CIA is unknown. We suggest that high levels of UDP and high expression of P2Y6 cooperatively promote RA pathogenesis.

UDP is an important extracellular nucleotide signaling molecule implicated in diverse biological processes via specific activation of metabotropic pyrimidine and purine nucleotide receptors (P2Y receptors). Pyrimidine and purine metabolism are components of nucleotide metabolism. Leflunomide, a selective inhibitor of de novo pyrimidine synthesis that alters pyrimidine metabolism, has been successfully used to treat RA by accelerating the metabolism of UDP to abolish the UDP adverse effect in RA (Breedveld et al., 2000; Fragoso et al., 2015). These results support our finding indicating the important role of UDP-related nucleotide metabolism in RA.

MRS2578 was found to inhibit the release of IL-6 and IL-8/keratinocyte chemoattractant by lung epithelial cells *in vivo*, whereas intrapulmonary application of the P2Y6 receptor agonist UDP increased the bronchoalveolar levels of IL-6 and keratinocyte chemoattractant. In addition, selective activation of P2Y6 receptors was found to induce the secretion of IL-6 and/or keratinocyte chemoattractant/IL-8 by murine and human lung epithelial cells *in vitro* (Vieira et al., 2011). The application of pressure was found to induce IL-6 expression through the P2Y6 receptor in human dental pulp cells (Satrawaha et al., 2011).

In summary, this study showed that the level of UDP is increased in RA and CIA and that UDP stimulates cell proliferation, cell migration and IL-6 secretion in RA synovial fibroblast cells as well as IL-6 secretion in CIA rats. Additionally, P2Y6 expression was found to be increased in RA and CIA synovial tissues. Treatment with the P2Y6 antagonist MRS2578 inhibited the effects of UDP on RA fibroblast cells and CIA. These results suggest that UDP is highly expressed in RA and stimulates RA pathogenesis by promoting P2Y6 activities to increase IL-6 production.

## Data Availability

The original contributions presented in the study are included in the article/Supplementary Material, further inquiries can be directed to the corresponding authors.
