# Supplementary material for: Uridine Diphosphate Promotes Rheumatoid Arthritis Through P2Y6 Activation
Source: Front Pharmacol. 2021 Apr 19;12:658511. doi: 10.3389/fphar.2021.658511 (PMC8089376; doi:10.3389/fphar.2021.658511)
Supplement: Supplementary file 1 [file table1.doc]

**Supplementary Table 1. Clinical information of patients donating synovial tissues and fluids**

| **RA** | | | | | | | **OA** | | | | | | | |
| --- | --- | --- | --- | --- | --- | --- | --- | --- | --- | --- | --- | --- | --- | --- |
| **NO.** | **Gender** | **Age** | **RF (IU)** | **Anti-CCP (U)** | **Agents** | **Sample**  **type** | **NO.** | **Gender** | **Age** | **K-Lscale** | **RF (IU)** | **Anti-CCP (U)** | **Agents** | **Sample**  **type** |
| R41 | ♀ | 42 | 64 | 65 | D,T | ST, SF | O41 | ♂ | 45 | 4 | 6 | 4.8 | N, S | ST, SF |
| R42 | ♀ | 81 | 162 | 101 | D,N | ST, SF | O42 | ♂ | 59 | 2 | - | 0.4 | N,T | ST, SF |
| R43 | ♀ | 51 | 134 | 86 | D, T | ST, SF | O43 | ♀ | 52 | 3 | 3.6 | 2.9 | S | ST, SF |
| R44 | ♀ | 59 | 30 | 54 | D | ST, SF | O44 | ♀ | 76 | 2 | 0.1 | 0.6 | N | ST, SF |
| R45 | ♀ | 21 | 67 | 91 | T | ST, SF | O45 | ♀ | 62 | 3 | 3.9 | 4.5 | T | ST, SF |
| R46 | ♀ | 48 | 86 | 75 | D, T | ST, SF | O46 | ♂ | 62 | 3 | 1.1 | 1.2 | N,S | ST, SF |
| R47 | ♀ | 58 | 56 | 46 | D | ST, SF | O47 | ♂ | 55 | 3 | 1.2 | 0.8 | T | ST, SF |
| R48 | ♀ | 62 | 78 | 46 | D, N | ST, SF | O48 | ♂ | 73 | 4 | 3.6 | 3.2 | S | ST, SF |
| R49 | ♀ | 65 | 361 | 73 | D | ST, SF | O49 | ♀ | 65 | 2 | 0.3 | - | N | ST, SF |
| R50 | ♀ | 67 | 329 | 130 | N | ST, SF | O50 | ♀ | 72 | 3 | 0.5 | 1.4 | N | ST, SF |

Anti-CCP: anticyclic citrullinated peptide; D: DMARDs, disease-modifying anti-rheumatic drugs; K-L grading scale: Kellgren-Lawrence grading scale; Female: ♀; Male: ♂; N: NSAIDs, Nonsteroidal anti-inflammatory drugs; OA: osteoarthritis; PB: peripheral blood; RA: rheumatoid arthritis; Rheumatoid factor: RF; S: SYSADOAs, symptomatic slow-acting drugs for osteoarthritis; SF: synovial fluids; T: Traditional Chinese medicine; ST: synovial tissues; SF: synovial fluids
