# Supplementary material for: Uridine Diphosphate Promotes Rheumatoid Arthritis Through P2Y6 Activation
Source: Front Pharmacol. 2021 Apr 19;12:658511. doi: 10.3389/fphar.2021.658511 (PMC8089376; doi:10.3389/fphar.2021.658511)
Supplement: Supplementary file 2 [file table2.doc]

**Supplementary Table 2. Clinical information of the patients donating blood and synovial fluids**

| **Rheumatoid Arthritis (RA)** | | | | | | | | | **Osteoarthritis (OA)** | | | | | | **Healthy volunteers** | | | |
| --- | --- | --- | --- | --- | --- | --- | --- | --- | --- | --- | --- | --- | --- | --- | --- | --- | --- | --- |
| NO. | Gender | | Age | UDP in SF  (ng/mL) | UDP in PB  (ng/mL) | RF (IU) | Anti-CCP (U) | Agents | NO. | Gender | Age | UDP in SF  (ng/mL) | K-Lscale | Agents | NO. | Gender | Age | UDP in PB  (ng/mL) |
| R1 | ♀ | 42 | | 9 | 150 | 64 | 65 | D | O1 | ♂ | 75 | 11 | 2.4 | N | H1 | ♂ | 34 | 26.6 |
| R2 | ♀ | 81 | | 43 | 28 | 162 | 101 | D | O2 | ♂ | 62 | 9 | 2.3 | N | H2 | ♀ | 43 | 30.8 |
| R3 | ♀ | 51 | | 20 | 24 | 134 | 86 | D, T | O3 | ♂ | 42 | 8.1 | 2.7 | S | H3 | ♀ | 46 | 31.3 |
| R4 | ♀ | 59 | | 23 | 27 | 30 | 54 | D | O4 | ♀ | 46 | 7.2 | 2.8 | N, S | H4 | ♂ | 52 | 2.64 |
| R5 | ♀ | 21 | | 51 | 32 | 67 | 91 | D, T | O5 | ♂ | 67 | 6.7 | 2.6 | T | H5 | ♀ | 71 | 28.16 |
| R6 | ♀ | 48 | | 16 | 186 | 86 | 75 | D | O6 | ♀ | 58 | 2.3 | 3.6 | N,S | H6 | ♂ | 63 | 24.42 |
| R7 | ♀ | 58 | | 18 | 26 | 56 | 46 | D | O7 | ♀ | 45 | 1.7 | 3.2 | S | H7 | ♀ | 44 | 31.14 |
| R8 | ♀ | 62 | | 19 | 31 | 78 | 46 | D | O8 | ♂ | 69 | 3.2 | 3.3 | N | H8 | ♂ | 57 | 25.98 |
| R9 | ♀ | 65 | | 62 | 33 | 361 | 73 | D | O9 | ♀ | 62 | 1.3 | 2.5 | N | H9 | ♂ | 42 | 21.95 |
| R10 | ♀ | 67 | | 58 | 44 | 329 | 130 | D, N | O10 | ♀ | 54 | 5.6 | 2.8 | N | H10 | ♂ | 31 | 15.49 |
| R11 | ♀ | 58 | | 22 | 31 | 152 | 78 | D | O11 | ♀ | 58 | 3.1 | 3.1 | S | H11 | ♂ | 36 | 27.77 |
| R12 | ♀ | 54 | | 25 | 28 | 153 | 132 | D | O12 | ♂ | 53 | 21.4 | 2.5 | S | H12 | ♀ | 48 | 48.77 |
| R13 | ♀ | 45 | | 22 | 29 | 78 | 64 | D | O13 | ♀ | 65 | 18.4 | 2.3 | S | H13 | ♂ | 23 | 18.90 |
| R14 | ♀ | 25 | | 27 | 30 | 54 | 85 | D, T | O14 | ♀ | 66 | 14.3 | 2.8 | S | H14 | ♂ | 36 | 22.26 |
| R15 | ♂ | 78 | | 6 | 115 | 53 | 39 | D | O15 | ♂ | 52 | 7.8 | 3.2 | N,S | H15 | ♂ | 45 | 19.07 |
| R16 | ♀ | 74 | | 100 | 22 | 759 | 157 | D, N | O16 | ♀ | 70 | 5.2 | 2.7 | N,S | H16 | ♂ | 38 | 17.25 |
| R17 | ♀ | 68 | | 120 | 24 | 724 | 201 | D, T | O17 | ♂ | 51 | 5.5 | - | N,S | H17 | ♀ | 42 | 27.05 |
| R18 | ♀ | 61 | | 28 | 25 | 59 | 63 | D, N | O18 | ♀ | 64 | 3.2 | 3.1 | N,S | H18 | ♀ | 55 | 31.24 |
| R19 | ♀ | 38 | | 23 | 26 | 121 | 56 | D | O19 | ♀ | 58 | 6.45 | 3.2 | N,S | H19 | ♀ | 63 | 67.37 |
| R20 | ♂ | 63 | | 11 | 151 | 77 | 81 | D, N | O20 | ♂ | 72 | 4.87 | 2.7 | N,S,T | H20 | ♂ | 57 | 20.33 |
| R21 | ♂ | 51 | | 10 | 28 | 87 | 77 | D | O21 | ♂ | 66 | 4.2 | 2.6 | N,S | H21 | ♀ | 48 | 28.13 |
| R22 | ♀ | 54 | | 110 | 25 | 753 | 82 | D | O22 | ♀ | 64 | 0.3 | 2.7 | T | H22 | ♀ | 68 | 26.78 |
| R23 | ♀ | 68 | | 97 | 27 | 631 | 106 | D | O23 | ♀ | 53 | 0.5 | 3.1 | N,S | H23 | ♀ | 27 | 30.78 |
| R24 | ♀ | 54 | | 27 | 31 | 213 | 111 | D, T | O24 | ♀ | 55 | 0.3 | 2.7 | N | H24 | ♀ | 31 | 31.29 |
| R25 | ♀ | 38 | | 77 | 24 | 436 | 67 | D | O25 | ♂ | 48 | 12.3 | 2.4 | S | H25 | ♂ | 38 | 2.64 |
| R26 | ♀ | 54 | | 64 | 24 | 365 | 137 | D, N | O26 | ♂ | 66 | 4 | 2.6 | - | H26 | ♀ | 45 | 26.25 |
| R27 | ♂ | 62 | | 25 | 22 | 243 | 87 | D, N | O27 | ♀ | 54 | 3.1 | - | S | H27 | ♀ | 57 | 67.32 |
| R28 | ♀ | 66 | | 108 | 20 | 654 | 355 | D, N | O28 | ♀ | 63 | 1.2 | 2.5 | N,S | H28 | ♀ | 48 | 22.66 |
| R29 | ♀ | 72 | | 101 | 189 | 476 | 476 | D, N | O29 | ♀ | 39 | 0.5 | 3.3 | N,S | H29 | ♀ | 32 | 23.89 |
| R30 | ♀ | 54 | | 150 | 26 | 198 | 1200 | D, N | O30 | ♂ | 48 | 0.2 | 2.4 | N | H30 | ♀ | 26 | 24.38 |
| R31 | ♀ | 74 | | 165 | 31 | 327 | 853 | D, N | O31 | ♀ | 64 | 8.3 | 2.7 | N | H31 | ♀ | 34 | 31.47 |
| R32 | ♀ | 77 | | 103 | 33 | 565 | 568 | D, T | O32 | ♂ | 78 | 0 | 3.3 | S | H32 | ♀ | 26 | 31.10 |
| R33 | ♂ | 54 | | 96 | 44 | 634 | 464 | D, N | O33 | ♀ | 63 | 1.8 | 2.9 | N | H33 | ♀ | 47 | 26.03 |
| R34 | ♀ | 23 | | 107 | 31 | 579 | 550 | D, N | O34 | ♂ | 54 | 1.1 | 3.1 | N,S | H34 | ♂ | 43 | 21.87 |
| R35 | ♀ | 54 | | 20 | 28 | 89 | 221 | D, T | O35 | ♂ | 59 | 0.7 | - | N,S | H35 | ♂ | 56 | 15.45 |
| R36 | ♀ | 46 | | 94 | 29 | 142 | 346 | D, T | O36 | ♂ | 52 | 0.4 | 2.6 | N | H36 | ♀ | 34 | 28.04 |

Anti-CCP: anticyclic citrullinated peptide; D: DMARDs, disease-modifying anti-rheumatic drugs; K-L grading scale: Kellgren-Lawrence grading scale; Female: ♀; Male: ♂; N: NSAIDs, Nonsteroidal anti-inflammatory drugs; PB: peripheral blood; Rheumatoid factor: RF; S: SYSADOAs, symptomatic slow-acting drugs for osteoarthritis; SF: synovial fluids; T: Traditional Chinese medicine
