# Supplementary material for: Uridine Diphosphate Promotes Rheumatoid Arthritis Through P2Y6 Activation
Source: Front Pharmacol. 2021 Apr 19;12:658511. doi: 10.3389/fphar.2021.658511 (PMC8089376; doi:10.3389/fphar.2021.658511)
Supplement: Supplementary file 3 [file table3.doc]

| **No.** | **Metabolites** | **Ion mode** | | **m/z** | **Δppm** | **Formula** | **OA (n=10)** | **RA (n=10)** | **P value (t test)** | **FC** | **VIP** | **OR (95%CI)** | **P value**  **(OR)** |
| --- | --- | --- | --- | --- | --- | --- | --- | --- | --- | --- | --- | --- | --- |
| 1 | **2-Aminobenzoic acid** | | **POS** | **138.05** | **-3.22** | **C7H7NO2** | **90.28±77.89** | **495.80±370.51** | **0.025** | **5.5** | **1.39** | **1.011 (0.999-1.022)** | **0.065** |
| 2 | **2-Hydroxy-3-methylbutyric acid** | | **POS** | **119.07** | **4.83** | **C5H10O3** | **2.95±2.11** | **6.39±2.10** | **0.018** | **2.2** | **1.36** | **704.3 (0.1-4901577)** | **0.146** |
| 3 | **9,12,13-****TriHOME** | | **NEG** | **329.23** | **-1.76** | **C18H34O5** | **52.59±30.18** | **124.56±70.17** | **0.043** | **2.4** | **1.24** | **1.068 (0.998-1.143)** | **0.058** |
| 4 | **Creatinine** | | **POS** | **114.06** | **-3.12** | **C4H7N3O** | **1232±1132** | **2920±498** | **0.007** | **2.4** | **0.99** | **1.003 (0.999-1.007)** | **0.103** |
| 5 | **Cyclic GMP** | | **POS** | **368.03** | **5.93** | **C10H12N5O7P** | **6.70±3.70** | **14.55±3.08** | **0.0025** | **2.2** | **1.35** | **4.58E12** | **0.989** |
| 6 | **Epinephrine sulfate** | | **NEG** | **308.04** | **1.62** | **C9H13NO6S** | **268.21±120.77** | **597.23±160.59** | **0.0025** | **2.2** | **1.51** | **2.186 (0-1.198E44)** | **0.988** |
| 7 | **Guanidinosuccinic acid** | | **POS** | **176.06** | **-6.33** | **C5H9N3O4** | **12.64±6.43** | **28.01±9.99** | **0.010** | **2.2** | **1.67** | **8.654E12** | **0.981** |
| 8 | **Guanosine** | | **POS** | **306.08** | **-1.52** | **C10H13N5O5** | **117.56±64.87** | **361.20±237.92** | **0.036** | **3.1** | **1.67** | **1.013 (0.999-1.027)** | **0.080** |
| 9 | **Isobutyryl-L-carnitine** | | **POS** | **232.15** | **-2.13** | **C11H21NO4** | **514.63±374.04** | **1073.99±446.96** | **0.040** | **2.1** | **1.36** | **1.005 (1.000-1.010)** | **0.037** |
| 10 | **Methionine sulfoxide** | | **POS** | **188.03** | **-2.71** | **C5H11NO3S** | **18.08±10.28** | **52.40±27.93** | **0.018** | **2.9** | **1.34** | **1.859 (0.504-6.864)** | **0.352** |
| 11 | **N-Acetyl-L-tyrosine** | | **POS** | **241.11** | **-11.27** | **C11H13NO4** | **6.89±4.13** | **14.59±5.88** | **0.025** | **2.1** | **1.31** | **1.751 (0.910-3.366)** | **0.093** |
| 12 | **Norepinephrine sulfate** | | **NEG** | **294.02** | **1.52** | **C8H11NO6S** | **100.69±44.06** | **212.92±44.31** | **0.001** | **2.1** | **1.51** | **9233 (0-6.9E219)** | **0.971** |
| 13 | **Phenol** | | **NEG** | **93.03** | **-9.13** | **C6H6O** | **1521±1208** | **3859±1928** | **0.030** | **2.5** | **1.47** | **1.001 (1-1.002)** | **0.025** |
| 14 | **Phenylpropanolamine** | | **POS** | **169.13** | **-0.069** | **C9H13NO** | **2.39±2.93** | **7.56±1.05** | **0.002** | **3.2** | **1.42** | **1.167E6 (0-4.5E21)** | **0.446** |
| 15 | **S-Adenosylhomocysteine** | | **POS** | **385.13** | **3.185** | **C14H20N6O5S** | **23.22±14.78** | **66.79±43.55** | **0.00049** | **2.8** | **1.41** | **1.130 (0.992-1.287)** | **0.067** |
| 16 | **Tauroursodeoxycholic acid** | | **NEG** | **498.29** | **2.09** | **C26H45NO6S** | **177.12±169.48** | **670.29±455.30** | **0.032** | **3.8** | **1.33** | **1.006 (1.001-1.010)** | **0.018** |
| 17 | **Uridine diphosphate** | | **NEG** | **402.99** | **0.045** | **C9H14N2O12P2** | **2.04±3.11** | **10.64±4.72** | **0.0039** | **5.2** | **1.54** | **2.314 (1.021-5.243)** | **0.044** |
| 18 | **Pyrophosphate** | | **POS** | **174.92** | **12.73** | **O7P2-4** | **29.13±30.42** | **0±0** | **0.041** | **0.5** | **1.61** | **0.286** | **0.997** |

**Supplementary table 3. Differential metabolites in synovial fluids**
