# Supplementary figures and images for: Uridine Diphosphate Promotes Rheumatoid Arthritis Through P2Y6 Activation
Source: Front Pharmacol. 2021 Apr 19;12:658511. doi: 10.3389/fphar.2021.658511 (PMC8089376; doi:10.3389/fphar.2021.658511)

**A**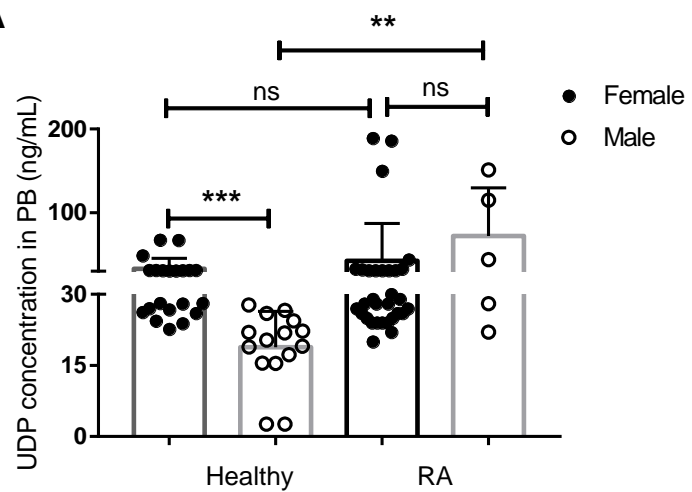**B**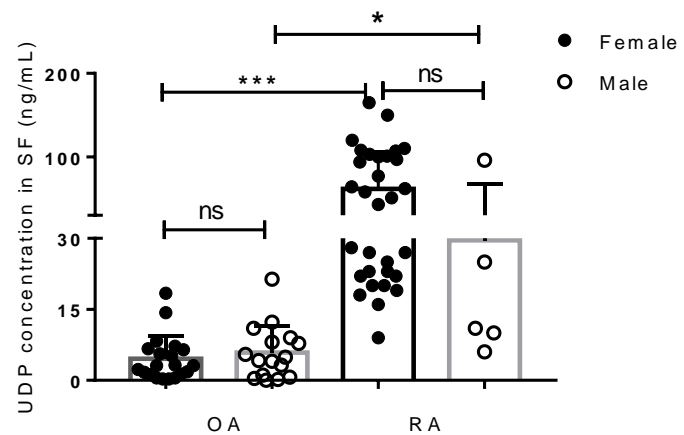

Supplement: Supplementary file 4 [file image1.pdf]

**A**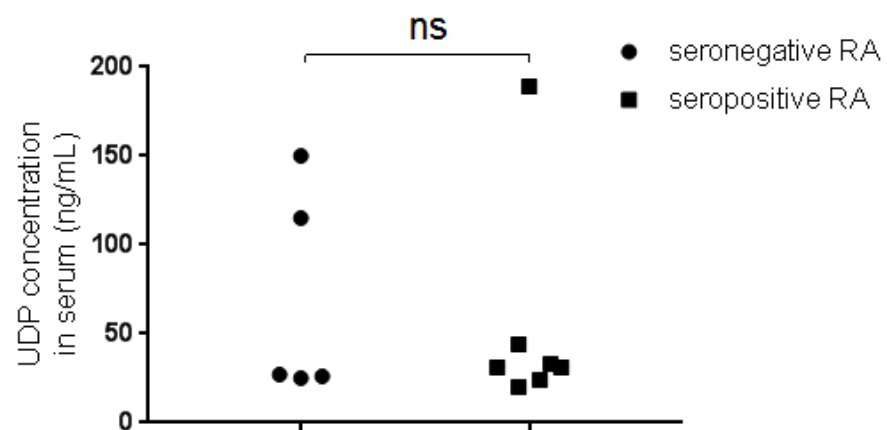**B**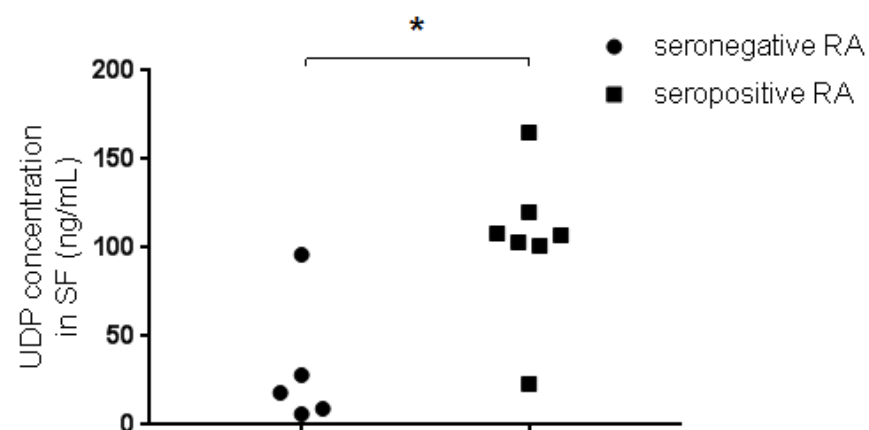

Supplement: Supplementary file 5 [file image2.pdf]
